# Beyond Steroids: Mycophenolate Mofetil Dosing in Immune Checkpoint Inhibitor-Mediated Hepatitis

**DOI:** 10.14740/jmc5381

**Published:** 2026-10-02

**Authors:** Shreya Shambhavi, Aksa Alina Joy, Emilia Wilk, Chirag Shah

**Affiliations:** a Rutgers Health/Community Medical Center, Toms River, NJ, USA

**Keywords:** Mycophenolate, Immune checkpoint inhibitor-induced hepatitis, Corticosteroids

## Abstract

Hepatotoxicity associated with immune checkpoint inhibitors (ICIs) is commonly characterized by elevation of serum aminotransferases, often leading to immunotherapy interruption affecting outcomes in cancer. Low-grade hepatitis usually responds to corticosteroids, but high-grade hepatitis often requires higher doses of steroids or additional immunosuppressants such as mycophenolate mofetil (MMF). With limited literature on MMF dosage and tapering, we present a case series of three patients who developed ICI-mediated hepatitis (ICH), ranging from grade 2 to 4, and were successfully treated with MMF along with concurrent low-dose corticosteroids. In all three cases, alternative etiologies, including viral hepatitis, auto-immune hepatitis, biliary pathologies, and hepatotoxic medications, were ruled out. Corticosteroids were initiated as first-line therapy for all patients; however, when the serological response remained minimal after the first 3 days, MMF was added at a dose ranging from 500 to 1,000 mg twice daily. Liver enzymes demonstrated a measurable decline within the first week of MMF initiation, with complete normalization achieved within 4–12 weeks during which MMF was gradually tapered. Two out of three patients experienced hepatitis relapse, with one following MMF cessation and another upon ICI rechallenge, both of whom responded successfully to MMF re-initiation at lower maintenance doses; two out of three patients had diarrhea, which was managed conservatively and one out of three patients had mild leukopenia, which was observed closely. MMF is a potent alternative for high-dose corticosteroids with a very manageable adverse effect profile and helps us avoid the severe side effects related to steroids, notably hyperglycemia, psychosis, infections, and poor outcomes in patients diagnosed with cancer. In one patient, hepatitis recurred after stopping MMF which was managed with re-initiation of MMF, which suggests that liver enzymes should be monitored on low-dose MMF even after their normalization rather than hastily stopping it. Moving forward, studies with larger sample sizes are needed to establish guidelines regarding MMF dosing and its tapering.

## Introduction

Immune checkpoint inhibitor (ICI)-mediated hepatitis (ICH) is an immune-related adverse event caused by cytotoxic T-lymphocyte-associated protein-4 (CTLA-4) inhibitors and programmed death ligand-1 (PDL-1) inhibitors. The overall incidence of ICH is similar between CTLA-4 inhibitor (3–15%) and PDL-1 inhibitor (1–17%) with higher incidence of grade 3/4 hepatitis in CTLA-4 inhibitor recipients as compared to patients receiving PDL-1 inhibitors (1–20% vs. 3–5%) [1, 2].

Based on the American Society of Clinical Oncology (ASCO) guidelines, depending on the severity of grades of hepatitis, the treatment for ICH is to temporarily or permanently discontinue ICIs and treat with corticosteroids and mycophenolate mofetil (MMF) [3]. Some recent reviews have reported the array of modalities used in the treatment of ICH including MMF, cyclosporine, antithymocyte globulin, and infliximab with varying degrees of efficacy and the time lags for the duration of response [4]. Although there are ASCO guidelines for dosing and weaning of steroids for ICH, there are no well-defined or standardized protocols regarding the dosing of MMF. In addition, existing literature on the maintenance dose and tapering of MMF remains notably limited and predominantly rests on the practitioner’s discretion. Henceforth, we present three cases primarily focused on the successful dosing and tapering of MMF in the treatment of ICH, along with dose strategies to prevent relapse of ICH and the management of relapsed ICH based on the pharmacokinetics of MMF.

The pathophysiology of ICH results is multifactorial, influenced by a variety of contributing factors. ICIs can cause clonal expansion of CD8^+^ T cells and upregulation of T-helper cells (TH1 and TH17), which might further increase inflammation through cytokine release (interleukin (IL)-2, tumor necrosis factor), eventually leading to the activation of cytotoxic T lymphocytes and innate immune cells such as macrophages [5–7]. In addition, ICIs are known to reduce regulatory T cells which are primarily responsible for secreting anti-inflammatory cytokines such as IL-10 and transforming growth factor-beta (TGF-β) [8]. The interplay of both phenomena can lead to an increased inflammatory state. It can also be attributed to T cells recognizing shared antigens between cancer and liver cells, leading to hepatitis in the course of destruction of cancer cells. PDL-1 and CTLA-4 receptors present on hepatic stellate cells and T-regulatory cells respectively are known to downregulate the effector T cells, leading to a steady state of immune tolerance at baseline [9]. ICIs by inhibiting the PDL-1 and CTLA-4 receptors, overcome the immune tolerance and eventually upregulate the inflammatory cascade through interferon gamma (IFN-γ) and granzymes as a result of increased multiplication of CD8^+^ T cells.

## Case Reports

This case series describes patients with ICH of different grades after receiving ICIs. ICH was defined and graded according to the Common Terminology Criteria for Adverse Events (CTCAE) 2017 guidelines (v5.0) [10].

### Case 1

The first case presents a 75-year-old female with metastatic adenocarcinoma of the lung who received four cycles of pembrolizumab (200 mg intravenous (IV) infusion every 3 weeks). After the fourth cycle, the patient experienced grade 4 hepatitis with serum alanine aminotransferase (ALT) of 800 U/L (normal range, 7–40 U/L), aspartate aminotransferase (AST) of 577 U/L (normal range, 0–30 U/L), and alkaline phosphatase (ALP) of 763 IU/L (normal range, 45–117 U/L). Ultrasound of the liver did not reveal any liver metastatic lesions, gallstones or gallbladder inflammation. No serological evidence of viral or auto-immune hepatitis was found. The patient was not concurrently on any other hepatotoxic drugs. Pembrolizumab was stopped immediately and oral prednisone was initiated at 2 mg/kg/day. Following a suboptimal response to corticosteroids alone over a duration of 3 days, the regimen was augmented with MMF at 1,000 mg administered orally twice daily. Notable improvements in serum aminotransferase levels were observed within the first week following initiation of MMF, with values recorded at ALT 130 U/L, AST 29 U/L, and ALP 188 IU/L and over a period of 6 weeks, culminating in ALT 36 U/L, AST 16 U/L, and ALP 77 IU/L. Liver enzymes ultimately normalized over a period of 12 weeks (ALT 19 U/L, AST 12 U/L, and ALP 156 IU/L). In this duration, MMF was weaned at 1,000 mg in the morning and 500 mg in the evening for 6 weeks, followed by 500 mg twice daily for 4 weeks and then to 250 mg twice daily over the next 2 weeks. Following the cessation of MMF, liver enzymes showed an upward trend even when the patient was maintained on a daily low-dose oral prednisone of 10 mg (ALT 50 U/L, AST U/L, ALP 385 IU/L). Due to the patient’s relapse of ICH with grade 2 hepatitis, MMF at 250 mg twice daily was re-initiated orally, following which the liver enzymes normalized over 3 weeks (ALT 17 U/L, AST 13 U/L, and ALP 126 IU/L). MMF was continued at a dose of 250 mg twice daily for another 3 weeks after normalization of liver enzymes. The graphical representation of the trends in the concentration of liver enzymes is summarized in Figure 1. Of note, the patient experienced few episodes of diarrhea intermittently which were managed conservatively, and had serological evidence of mild leukopenia of 3,300/µL.

**Figure 1:**
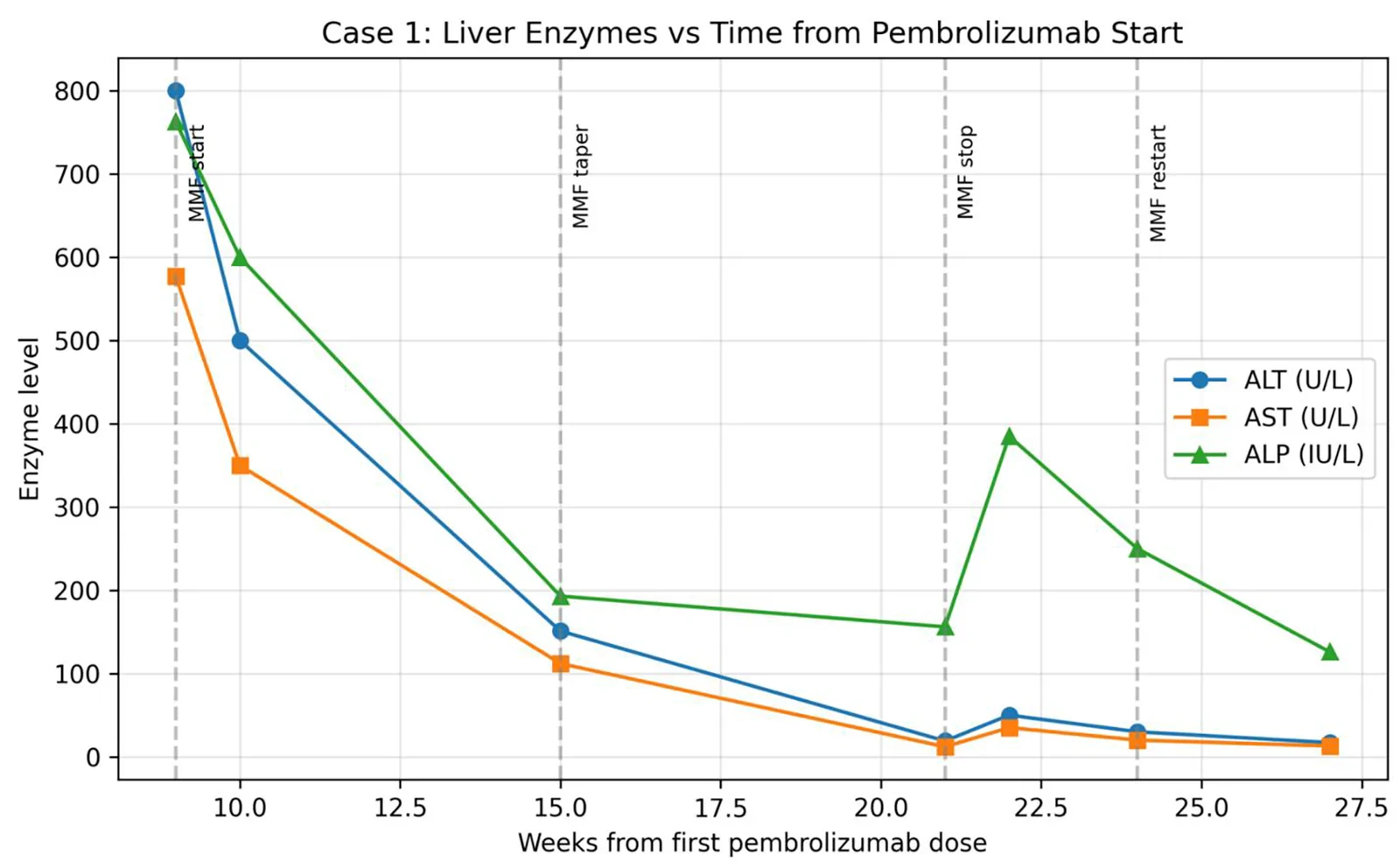
Graphical representation of trends of different liver enzymes in case 1 in relation to their response to MMF. MMF: mycophenolate mofetil.

### Case 2

The second case presents an 81-year-old female with squamous cell carcinoma of the lung. She was on atezolizumab intravenously at a dose of 1,200 mg infusion every 3 weeks. After three cycles of atezolizumab, the patient’s liver enzymes began to increase with serum ALT 339 U/L, AST 88 U/L, and ALP 260 IU/L, qualifying for grade 3 ICH. Alternate common causes of transaminitis, including cholecystitis, viral hepatitis, autoimmune hepatitis, and concurrent hepatotoxic drug use, were ruled out. The hepatotoxic drug was held and oral prednisone was initiated at 2 mg/kg/day. With minimal improvement with corticosteroids alone for 3 days, MMF at a dose of 1,000 mg twice daily orally was added to the regimen. A decline in serum aminotransferase level was observed in the first week of initiation of MMF (ALT 130 U/L, AST 29 U/L, ALP 188 IU/L) and they normalized completely over a period of 6 weeks (ALT 36 U/L, AST 16 U/L, ALP 77 IU/L). During the course of resolution, MMF was weaned off at a dose of 1,000 mg in the morning and 500 mg in the evening for the first 6 weeks, followed by 500 mg twice daily for 6 weeks which was followed by 250 mg BID for the next 2 weeks at a maintenance therapy dose even after resolution to prevent relapse. The graphical representation of the trends in the concentration of liver enzymes in the second patient is summarized in Figure 2. The patient experienced 3–4 episodes of diarrhea between his fourth to eighth week of MMF consumption, which were managed through conservative measures.

**Figure 2:**
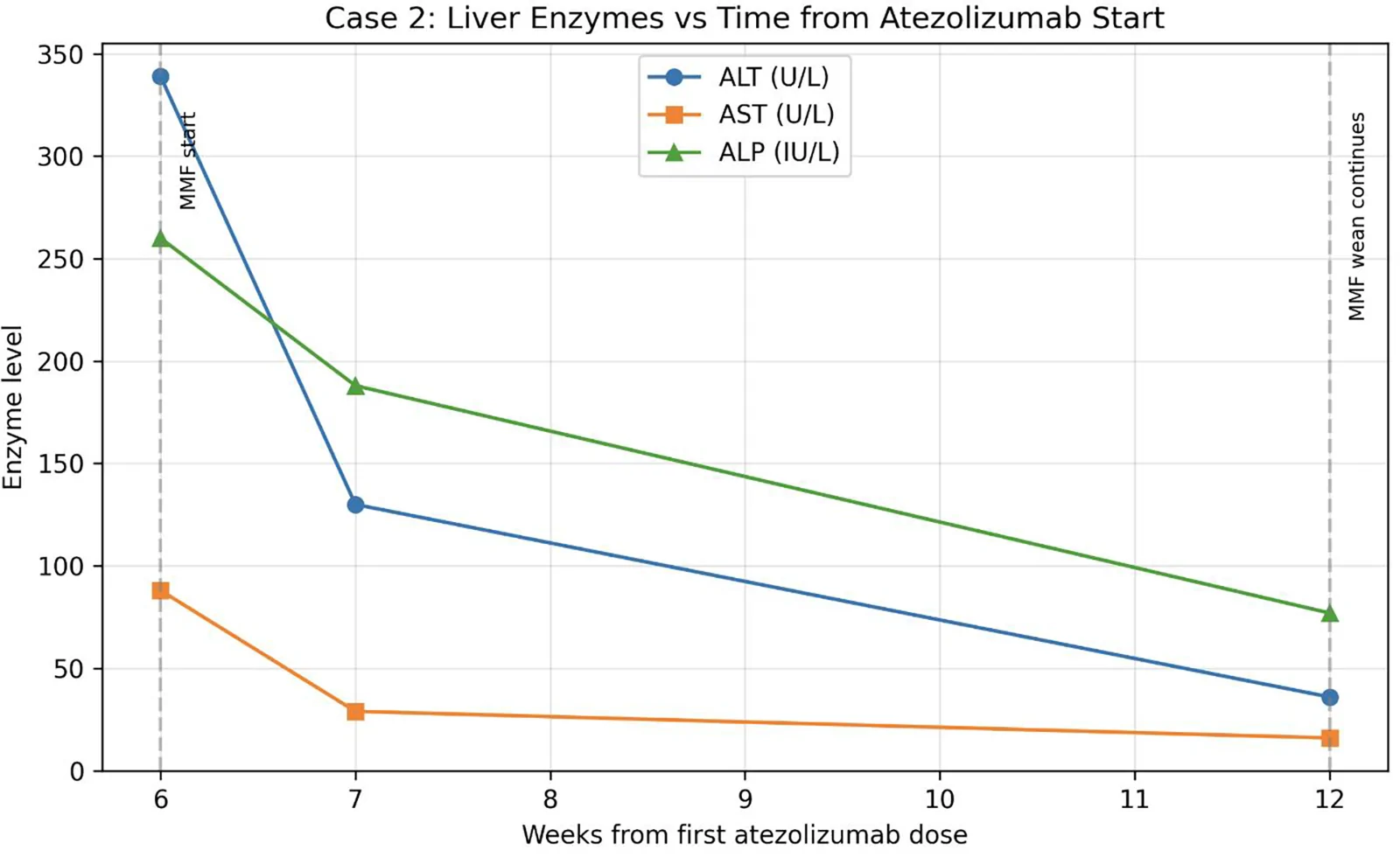
Graphical representation of trends of different liver enzymes in case 2 in relation to their response to MMF. MMF: mycophenolate mofetil.

### Case 3

The third case presents a 74-year-old male who was diagnosed with malignant melanoma. He was initiated on combination nivolumab 1 mg/kg and ipilimumab 3 mg/kg regimen every 3 weeks. After the second cycle of nivolumab and ipilimumab, the patient experienced grade 2 hepatitis with elevation in liver enzymes; serum ALT 130 U/L, AST 45 U/L, and ALP 67 IU/L. Ultrasound of the liver was unremarkable for liver metastases, gallstones, and gallbladder thickening. The viral hepatitis panel was also negative. Ipilimumab and nivolumab were held immediately and oral prednisone was initiated at the dose of 0.5 mg/kg/day after which serum levels of ALT and AST started to gradually decrease and they eventually normalized in the fourth week from initiation, with serum ALT 52 U/L and AST 30 U/L. The patient was slowly being weaned off corticosteroids. After 4–5 weeks of normalization of liver enzymes, nivolumab monotherapy was re-initiated, following which grade 2 hepatitis occurred despite the patient being on a daily low-dose prednisone of 5 mg (ALT 128 U/L, AST 27 U/L, ALP 333 IU/L). MMF was initiated at a dose of 500 mg twice daily orally and the patient’s liver enzymes reduced to normal range over the next 3 weeks (ALT 50 U/L, AST 27 U/L, ALP 71 IU/L). Following normalization of liver enzymes, MMF dosage was decreased to 250 mg twice daily and continued for another 2 weeks post-resolution of hepatitis. The graphical representation of the trends in the concentration of liver enzymes in the third patient is summarized in Figure 3.

**Figure 3:**
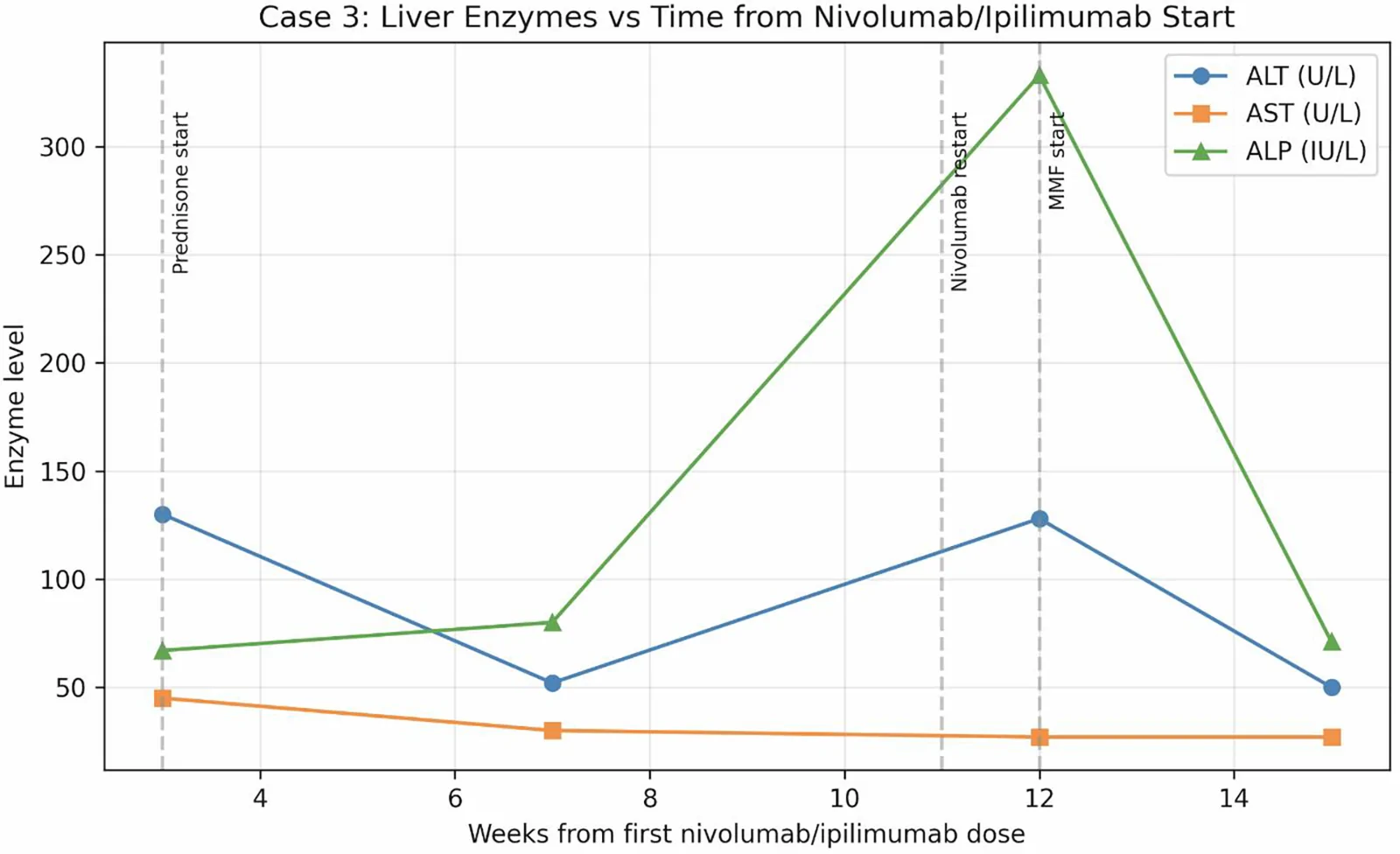
Graphical representation of trends of different liver enzymes in case 3 in relation to their response to MMF. MMF: mycophenolate mofetil.

### Pharmacokinetics of MMF and its dosing

MMF is the pro-drug of mycophenolic acid (MPA). MMF is metabolized by glucuronyl transferase to mycophenolic acid glucuronide (MPAG), which is pharmacologically inactive. MPAG is then converted to the active form as MPA during enterohepatic recirculation. Ultimately, it is excreted in the urine as the inactive metabolite MPAG [11].

As a result of enterohepatic recirculation, secondary peaks in plasma concentrations of MPAG/MPA are observed from 8 to 12 h post-dose [12]. Data reported in post-transplant patients show that, due to the non-stationarity of MPA pharmacokinetics, in the early period (first few weeks) the mean area under curves (AUCs) of MPA are approximately 30–50% lower compared to the late period. MMF has high oral bioavailability, reported to be 78–94%. Additionally, it has a rapid onset of action, with the MPA maximum plasma concentration (C_max_) observed from 0.5 to 1.5 h [12].

The high oral bioavailability of MMF around 78–94% and its T_max_ of 0.5–1.5 h (rapid absorption) suggest that each dose produces a brisk peak and that even partial dose changes can lead to a difference in plasma levels. If we abruptly stop MMF, it could lead to a steep fall in its peak and trough levels leading to sudden disequilibrium of concentration of MMF in the body and a lack of buffer dose of MMF.

Its time-dependent clearance (higher early, lower later) indicates that the same dose gives a higher AUC later in the course; therefore, a reduced dose can still yield meaningful serum levels during long-term maintenance (e.g. 250–500 mg BID). In addition, the enterohepatic recirculation of MMF adds a second wave of MPA exposure hours after dosing, extending the effective exposure period even if peak doses are reduced. Crucially, ionosine-5’-monophosphate dehydrogenase (IMPDH) inhibition is concentration-dependent and reversible, and the immunosuppressive effect correlates with MPA exposure (AUC) rather than a single trough concentration. This pharmacodynamic reversibility provides a mechanistic basis for graded dose reduction, whereby proportionate decrements in MPA exposure yield predictable reductions in lymphocyte suppression rather than an abrupt loss of immunosuppressive control. Altogether, keeping in consideration the pharmacokinetics and pharmacodynamics of MMF, it is suggested that if we adopt stepwise reductions in the dosage of MMF, as we did with our patients, we can create controlled decrements in MMF concentration leading to a steadier MPA exposure that the immune system can tolerate with lower toxicity risk.

### Adverse effects of MMF

The most common adverse reactions reported (20% or greater) include leukopenia (23–45%), anemia (25–42%), gastrointestinal (GI) adverse effects such as diarrhea (54–68%), and serious infections (12–27%) [13]. In the literature, 40–79% of patients have been described to require dose reduction or withdrawal of MMF due to intolerance of adverse effects, most commonly GI.

### Why mycophenolate over steroids

In a mouse melanoma model treated with immune checkpoint blockers, systemic corticosteroids have been shown to impair early memory CD8^+^ T-cell responses and reduce low-affinity memory CD8^+^ T cells, inhibit T-cell proliferation, blunt anti-tumor immunity, and promote tumor regrowth in mouse melanoma models treated with ICI [14, 15].

In addition, they have also been shown to inhibit dendritic cells, promote expression of inhibitory checkpoint receptors and IL-10, driving a dysfunctional and exhausted phenotype of CD8^+^ T cells, thereby compromising anti-tumor function [15].

On the other hand, MMF acts via inhibition of ionosine monophosphate dehydrogenase, preferentially targeting proliferating T and B lymphocytes, and is generally considered less broadly immunosuppressive at the genomic level than high-dose corticosteroids. Mechanistic analyses in auto-immune and transplant models show that MMF-containing regimens alter T-cell activation, trafficking, and effector functions without uniformly depleting CD8^+^ T-cell subsets, implying a more selective impact on proliferating effector cells rather than a global suppression [16, 17]. Preclinical studies suggest that MMF lowers cluster of differentiation (CD) co-stimulatory molecules (CD40, CD80, CD86), cytokines (IL-12, IL-18), and allostimulatory capacity, indirectly altering T-cell activation and polarization rather than direct global T-cell depletion [18]. In addition, it preferentially affects activated/proliferating lymphocytes, consistent with its ionosine monophosphate dehydrogenase-dependent mechanism [19]. The detailed comparison of the mechanism of action of mycophenolate and corticosteroids is summarized in Figure 4.

**Figure 4:**
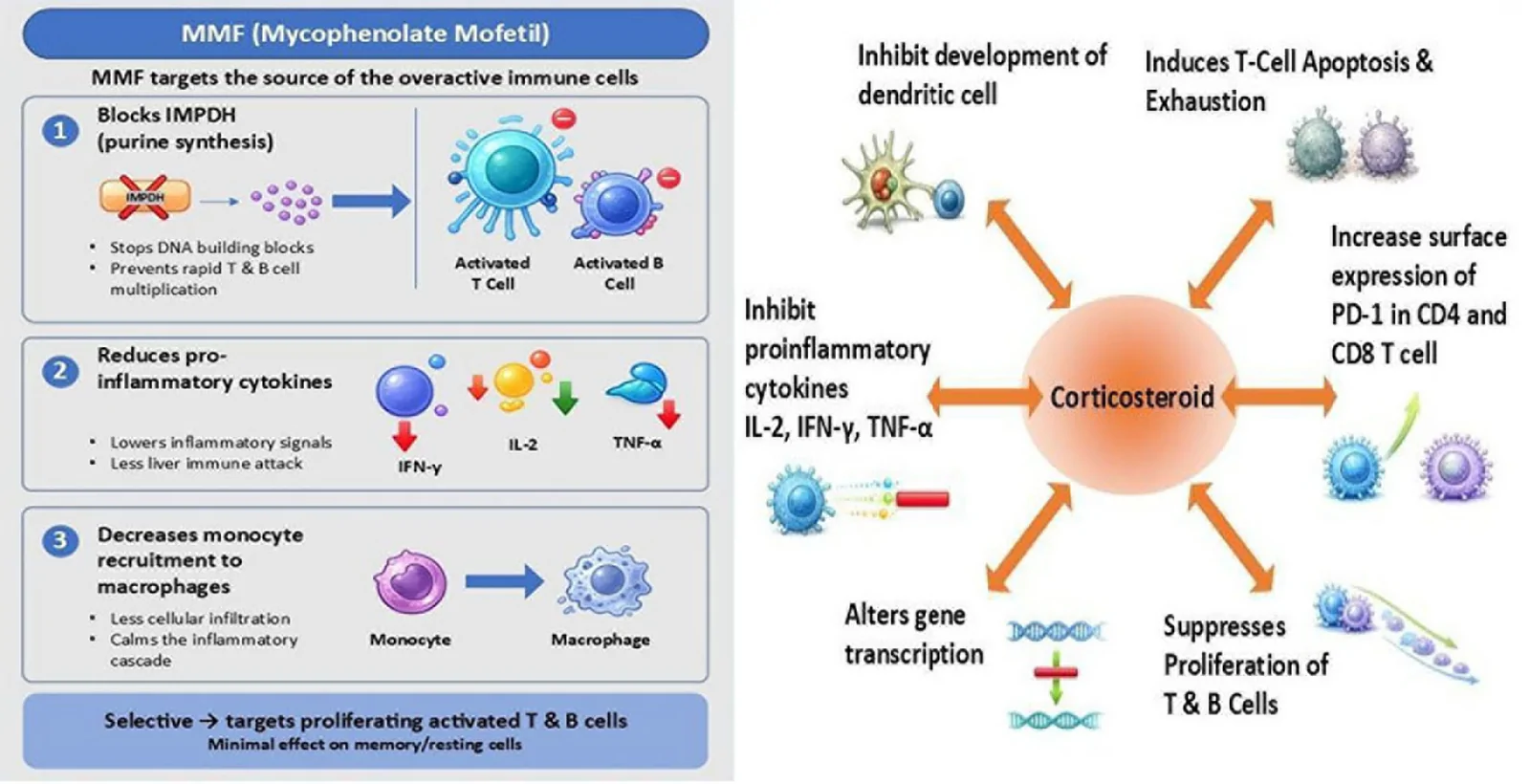
Diagrammatic representation of different modes of action of MMF (selective approach) vs. corticosteroids (global suppression). MMF: mycophenolate mofetil.

## Discussion

Previous studies have shown that the use of MMF with corticosteroids is more effective in treating ICH than corticosteroids alone. Fewer studies have described the dosage and tapering of MMF in patients with ICH [20–22]. Despite some description of the initial MMF dosage, the literature lacks clear, well-defined guidelines on dosing and treatment duration for preventing ICH relapse, which is among the most common reasons for immunotherapy failure. Moreover, there is limited literature on what needs to be done in terms of MMF continuation or cessation after normalization of liver enzymes. Our case series is the first of its kind, as we sought to address this gray zone in the literature on ICH and to prevent relapse of ICH with a steady tapering of MMF by reducing the dose by 500 mg over 4–6 weeks. We noticed that patients experienced a relapse of hepatitis when MMF was weaned off hastily, which can be attributed to its shorter half-life and rapid enterohepatic circulation in a recovering liver and suggests that patients should be monitored on low-dose MMF for a few more weeks even after normalization of liver enzymes, rather than its abrupt cessation. By following the approach mentioned above, we could prevent the relapse of ICH in our patients and prevent ICI interruption. There is mixed practice in the management of ICI-induced hepatitis relapse in patients already receiving low-dose steroid therapy. Although the ASCO guidelines recommend corticosteroids to be tapered over 4–6 weeks duration, clinicians commonly taper corticosteroids over a longer period and keep their patients on a daily low-dose maintenance of 5–10 mg/day of oral prednisone and there has been numerous cases of spontaneous ICH relapse or when rechallenged with ICI treatment even on low-dose steroids. We observed a similar pattern in our cohort, two out of our three patients showed relapse of ICH despite being on low-dose prednisone (precisely 10 mg/day). In such cases of ICH relapse on low-dose steroids, low-dose MMF (250–500 mg twice daily) proved to be an optimal dose for ≤ grade 2 hepatitis. While our patients did not experience high-grade (≥ grade 2) relapse of hepatitis, based on our observation in the low-grade hepatitis, we can postulate that high-dose MMF (1,000–2,000 mg twice daily) would most likely be effective in treating high-grade (≥ grade 2) relapse cases. We also concluded that MMF should be continued at a low dose (250 mg twice daily) for a few weeks (2–3 weeks) even after normalization of enzymes and patients should be monitored for that interval to prevent further relapse.

### Conclusions

Lastly, we opted for an approach that included prompt addition of MMF after a suboptimal response was observed with a decent dose of prednisone (1–2 mg/kg/day), rather than employment of pulse-dose corticosteroids (1,000 mg/day) before initiating MMF, as done in previous studies [21]. The rationale was the preferential targeting of proliferating effector T cells with MMF rather than the global suppression seen with corticosteroids and avoidance of serious adverse effects of high-dose steroids, which include severe hyperglycemia, increased risk of infections, loss of anti-tumor response and neuropsychiatric disturbances. The study has its own limitations. Firstly, the sample size is very small and it lacks racial variability as all three patients were Caucasian. Using this study as a base, larger studies and trials with higher sample size can help us yield robust and racially inclusive conclusions on the observations noted above in our case series. In conclusion, in today’s era where ICIs are the pillars of treatments of a plethora of solid and hematological malignancies, it is imperative to implement novel and selective strategies to mitigate ICI-mediated adverse events.

### Learning points

MMF is an effective steroid-sparing agent in ICH and can be added early (within 3–5 days) when suboptimal response is seen with prednisone 1–2 mg/kg/day, thereby avoiding the toxicity of pulse-dose corticosteroids.

MMF should be tapered gradually (by 500 mg every 4–6 weeks) rather than stopped abruptly, as its short half-life and enterohepatic circulation predispose a recovering liver to relapse after hasty cessation.

Low-dose MMF (250 mg twice daily) should be continued for 2–3 weeks after liver enzymes normalize, with ongoing monitoring, rather than being discontinued immediately on normalization.

For low-grade (≤ grade 2) relapse occurring on low-dose steroids, low-dose MMF (250–500 mg twice daily) is an effective option, and higher doses can be extrapolated to higher-grade relapse.

## Data Availability

All data generated or analyzed during this study are included in this published article.

## Abbreviations

ALP: alkaline phosphatase
ALT: alanine aminotransferase
ASCO: American Society of Clinical Oncology
AST: aspartate aminotransferase
AUCs: area under curves
BID: *bis in die*/two times daily
CD: cluster of differentiation
CTCAE: Common Terminology Criteria for Adverse Events
CTLA-4: cytotoxic T-lymphocyte-associated protein-4
C_max_: maximum plasma concentration
GI: gastrointestinal
ICH: immune checkpoint inhibitor-mediated hepatitis
ICIs: immune checkpoint inhibitors
IFN-γ: interferon gamma
IL: interleukin
IMPDH: ionosine-5’-monophosphate dehydrogenase
IV: intravenous
MMF: mycophenolate mofetil
MPA: mycophenolic acid
MPAG: mycophenolic acid glucuronide
PDL-1: programmed death ligand-1
TGF-β: transforming growth factor-beta
TH: T-helper cells
T_max_: time to attain maximum plasma concentration
µL: microliter
